# miR-302b is a potential molecular marker of esophageal squamous cell carcinoma and functions as a tumor suppressor by targeting ErbB4

**DOI:** 10.1186/1756-9966-33-10

**Published:** 2014-01-19

**Authors:** Mingxin Zhang, Qi Yang, Lingmin Zhang, Suna Zhou, Wenguang Ye, Qinglin Yao, Zongfang Li, Cheng Huang, Qinsheng Wen, Jingjie Wang

**Affiliations:** 1 Department of Gastroenterology, Tangdu Hospital, Fourth Military Medical University, Xi’an 710038 Shaanxi Province, China; 2 Department of Anesthesiology, First Affiliated Hospital, Medical School, Xi’an Jiaotong University, Xi’an 710061 Shaanxi Province, China; 3 Department of Radiotherapy, Tangdu Hospital, Fourth Military Medical University, Xi'an 710038 Shaanxi Province, China; 4 Department of General Surgery, Second Affiliated Hospital, School of Medicine, Xi’an Jiaotong University, Xi’an 710004Shaanxi Province, China; 5 Department of genetics and molecular biology, Medical school, Xi’an Jiaotong University/Key Laboratory of Environment and Genes Related to Diseases, Ministry of Education, Xi’an 710061Shaanxi Province, China

**Keywords:** miR-302b, ErbB4, Esophageal squamous cell carcinoma

## Abstract

**Background:**

ErbB4 expression has been noted in various tumors, but its regulatory mechanism in esophageal squamous cell carcinoma (ESCC) remains unclear. The aim of this study was to investigate whether miR-302b regulates the expression of ErbB4 at the post-transcriptional level and to determine its expression, significance, and function in ESCC.

**Methods:**

We used real-time reverse transcriptase-polymerase chain reaction to quantify the expression of miR-302b in 50 ESCC tissues and analyzed its relationship with clinicopathological factors and survival. Then, we investigated the post-transcriptional regulation of ErbB4 expression using immunoblot analysis and luciferase reporter assays. Finally, the effects of miR-302b on proliferation, apoptosis, and invasion of ESCC cells was detected using MTT, flow cytometric analysis, and transwell invasion assays, respectively.

**Results:**

miR-302b was significantly down-regulated and correlated with tumor differentiation and lymph node metastasis in ESCC. Univariate and multivariate analyses indicated that low miR-302b expression might be a poor prognostic factor. Further studies demonstrated that miR-302b post-transcriptionally down-regulated the expression of ErbB4 *in vitro*. Moreover, miR-302b inhibited proliferation by inducing apoptosis and repressed invasion in the ESCC cell lines.

**Conclusions:**

miR-302b is a potential molecular marker of ESCC and functions as a tumor suppressor by post-transcriptionally regulating ErbB4.

## Introduction

Esophageal squamous cell carcinoma (ESCC) is a highly aggressive neoplasm with geographic characteristics and poor prognosis. About one half of all ESCC cases in the world occur in China [1]. Over the past decades, more and more doctors have chosen to focus on the field of molecular targeted therapies [2]. One of the attractive targets in ESCC is the ErbB/HER subfamily, which regulates cellular proliferation, differentiation, and programmed cell death [3]. The ErbB/HER subfamily of receptor tyrosine kinases includes four members: EGFR (also known as ErbB1 or HER1), ErbB2 (c-Neu or HER2), ErbB3 (HER3), and ErbB4 (HER4) [4].

A multitude of studies have characterized the expression and significance of the HER family in ESCC [5-10]. EGFR and ErbB2 have been shown to be overexpressed in ESCCs compared to non-tumor tissues, and these proteins are important markers for the analysis of the prognosis and clinical course of the disease [5-8]. ErbB3 is also up-regulated in ESCC and correlates with a clinical response to chemotherapy, but it has a limited prognostic value for survival in ESCC [9,10]. ErbB4 is frequently up-regulated in various cancer tissues [11-15], and experimental down-regulation of ErbB4 in different tumor cells suppresses growth [16-20]. Xu et al. found that extranuclear ErbB4 had negative effects on the progression of ESCC, whereas the nuclear translocation of ErbB4 exhibited a tumor-promoting property [12]. Pang et al. reported that knockdown of ErbB4 inhibited migration and invasion of the ESCC cell line Eca-109 [16]. However, to our knowledge, the regulatory mechanism of ErbB4 in ESCC is largely unknown.

MicroRNAs (miRNAs) represent a class of small non-coding RNAs that regulate gene expression at the post-transcriptional level. Currently, emerging results have revealed that miRNAs are involved in cancer pathogenesis and can function as oncogenes or tumor suppressor genes [21]. miR-302b is a member of the miR-302 cluster, which regulates the regulatory circuitry controlling ES cell “stemness” [22]. Recently, it was found that the overexpression of miR-302b induced caspase-3-mediated apoptosis of the human neuroblastoma SH-SY5Y cell line [23].

Since miRNAs predicted to target a gene can be searched by online computational algorithm such as TargetScan (http://www.targetscan.org/vert_50/) or PicTar (http://pictar.mdc-berlin.de/). TargetScan and PicTar both predicted that ErbB4 is a target gene for miR-302b. Based on these observations, the aim of this study was to detect the expression of miR-302b in ESCC tissues and analyze its correlation with clinicopathological factors or prognosis, as well as to determine the post-transcriptional regulatory relationship between miR-302b and ErbB4. Furthermore, we examined whether manipulating the expression of miR-302b affected ESCC cell behaviors, which could provide a potential molecular therapeutic target for the treatment of human ESCC.

## Methods

### Patient samples and cell lines

Between January 2009 and December 2010, 60 patients received resection for ESCC at First Affiliated Hospital, Medical School, Xi’an JiaoTong University. Of these, the tumor staging, clinicopathological information, or follow up was incomplete for 10 patients. As a result, 50 patients were retrospectively reviewed. None of these 50 patients received neoadjuvant therapy before operation. Fresh cancer tissues and paired normal adjacent tissues (NAT) were obtained from these patients. The differentiation grade, TNM stage, and lymph node status were classified according to the UICC/AJCC TNM classification (seventh edition). The Institutional Ethics Committee approved this project and written informed consents were obtained from the patients. The ESCC cell lines (Eca109, Ec9706, and TE-1) and esaphagel normal cell line (Het-1A) were obtained from the Cell Bank of Shanghai (China) and cultured in RPMI 1640 medium supplemented with 10% fetal bovine serum (FBS), 100 units/mL penicillin, and 100 g/mL streptomycin at 37°C in a 5% CO_2_ incubator.

### Quantitative reverse transcription-PCR (qRT-PCR) for mature miRNA

qRT-PCR was carried out using the PrimeScript® RT reagent Kit (Perfect Real Time) and a BioRad iQ5 Real-Time PCR Detection System. The reverse transcription reaction was carried out in a 20 μL volume with 1 μg total RNA. The reaction was incubated at 37°C for 15 min, then 85°C for 5 sec; 1 μL of the RT product was used in each PCR. The PCR cycling began with template denaturation at 95°C for 5 min, followed by 40 cycles of 95°C for 10 sec, 60°C for 20 sec, and 72°C for 20 sec. U6 snRNA levels were used for normalization. The following primer sequences were used in this section: (1) ErbB4: random hexamers (RT primers), 5′-AGGAGTGAAATTGGACACAGC-3′ (forward primer for qRT-PCR), and 5′-TCCATCTCGGTATACAAACTGGT-3′ (reverse primer for qRT-PCR); (2) miR-302b: 5′- GTCGTATCCAGTGCGTGTCGTGGAGTCGGCAATTGCACTGGA TACGACCTACTAA -3′ (RT primer), 5′-GATAAGTGCT TCCATGT-3′ (forward primer for qRT-PCR), and 5′-CAGTGCGTGTCGTGGAGT- 3′ (reverse primer for qRT-PCR); (3) U6: 5′-CGCTTCACGAATTTGCGTGTCAT- 3′ (RT primer), 5′-GCT TCGGCAGCACATATACTAAAAT-3′ (forward primer for qRT-PCR), and 5′-CGCT TCACGAATTTGCGTGTCAT-3′ (reverse primer for qRT-PCR). A control reaction without reverse transcriptase was included, and the lack of signal from this reaction ensured that there was no genomic DNA contamination. In addition, the final PCR products were resolved using agarose gel electrophoresis, and a single band of the expected size indicated the specificity of the reaction. The expression levels relative to U6 were calculated using the formula 2^-ΔΔCT^.

### Immunoblot analysis

For immunoblot analyses, 20 μg total proteins were electrophoresed on a 10% SDS-PAGE gel, transferred to PVDF membrane, blocked, and then incubated with primary antibody. The blots were then incubated with the corresponding horseradish peroxidase (HRP)-conjugated secondary antibody at room temperature for 2 h. Then, the membranes were visualized by exposure to X-ray film in dark following a chemiluminescence reaction using the enhanced ECL detection reagents (Amersham, Little Chalfont, Buckinghamshire, England) according to the manufacturer’s instructions. Densitometry analysis was performed using the Scion Image software.

### Plasmid construction and cell transfection

The sequence of the precursor miR-302b was synthesized and cloned into the pcDNA™6.2-GW/EmGFP-miR expression vector (Invitrogen, Carlsbad, CA, USA). The ErbB4 3′-UTR target site sequence and the sequence containing the mutation of three bases in the miR-302b target site were synthesized and cloned downstream of the luciferase gene in the pmirGLO luciferase vector (Promega, Madison, WI, USA). All procedures were performed as previously described [24]. These vectors were named miR-302b, ErbB4-WT, and ErbB4-MT, respectively. All constructs were sequenced. The anti-miR-302b inhibitor (2′–O-methyl antisense oligonucleotide, anti-miR-302b) and the anti-miR-inhibitors-Negative control (2′–O-methyl scrambled miRNA, control) were purchased from AngRang Inc. (Xi’an, China). Cell transfection was performed using Lipofectamine 2000 (Invitrogen, Carlsbad, CA, USA) according to the manufacturer’s protocol. Total RNA and protein were prepared 48 h after transfection and were used for qRT-PCR or immunoblot analysis, respectively. The DNA fragment sequences are listed in Table 1.

**Table 1:** Oligonucleotides used for plasmid construction

**Name**	**Sequence**
pre-miR-302b-top	5*'*-AATTCGCTCCCTTCAACTTTAACATGGAAGTGCTTTCTGTGACTTTAAAAGTAAGTGCTTCCATGTTTTAGTAGGAGTA-3*'*
pre-miR-302b-bottom	5*'*-AGCTTACTCCTACTAAAACATGGAAGCACTTACTTTTAAAGTCACAGAAAGCACTTCCATGTTAAAGTTGAAGGGAGCG-3*'*
Erbb4-WT-sense	5*'*-CGAATTCACTCAGAAATGTAGTTT*GCACTT*AAGCTGTAATTTTATTTGTTC-3*'*
Erbb4-WT-antisense	5*'*-TCGAGAACAAATAAAATTACAGCTTAAGTGCAAACTACATTTCTGAGTGAATTCGAGCT-3*'*
Erbb4-MT-sense	5*'*-CGAATTCACTCAGAAATGTAGTTT*GGTGTT*AAGCTGTAATTTTATTTGTTC-3*'*
Erbb4-MT-antisense	5*'*-TCGAGAACAAATAAAATTACAGCTTAACACCAAACTACATTTCTGAGTGAATTCGAGCT-3*'*

### Luciferase assay

The cells were co-transfected with ErbB4-WT or ErbB4-MT and miR-302b or mock (pcDNA™6.2-GW/EmGFP-miR). Luciferase activity was measured 24 h after transfection using the Dual-Glo luciferase assay system (Promega, Madison, WI, USA). All experimental protocols were performed according to the manufacturer’s instructions. The normalized firefly luciferase activity (firefly luciferase activity/*Renilla* luciferase activity) for each construct was compared to that of the pmirGLO Vector no-insert control.

### Cell viability assays

Cell viability was determined using an MTT assay according to the manufacturer’s protocol. pcDNA™6.2-GW/EmGFP-miR (mock) and anti-miR-inhibitors-Negative control (control) were used as the controls for miR-302b and anti-miR-302b, respectively. The absorbance of each well was measured using a multidetection microplate reader (BMG LABTECH, Durham, NC, USA) at a wavelength of 570 nm. All experiments were performed in quadruplicate.

### Cell apoptosis assays

Cells were washed with PBS and resuspended in 500 μL binding buffer containing 2.5 μL annexin V-phycoerythrin (PE) and 5 μL 7-amino-actinomycin D (7-AAD) to determine the phosphatidylserine (PS) exposure on the outer plasma membrane. After incubation, the samples were analyzed using flow cytometry (FACSCalibur, BD Biosciences, San Jose, CA). The experiment was repeated three times.

### Cell invasion assay

Cell invasion was measured using transwell chambers (Millipore, Billerica, USA) coated with Matrigel. After transfection, the harvested cells were suspended in serum free RPMI 1640 and were added into the upper compartment of the chamber; conditioned RPMI 1640 medium with 20% (v/v) FBS was used as a chemoattractant and placed in the bottom compartment of the chamber. After incubation, the cells were removed from the upper surface of the filter with a cotton swab. The invaded cells were then fixed and stained using 0.1% crystal violet. The cells were quantified from five different fields under a light microscope. The experiment was repeated in triplicate.

### Statistical analysis

To investigate the association of miR-302b expression with clinicopathological features and survival, miR-302b expression values were separated into low and high expression groups using the median expression value within the cohort as a cutoff. A Fisher’s exact text was used to analyze the relationship between miR-302b and the various clinicopathological characteristics. Progression-free survival (PFS) was defined as the time from the first day of treatment to the time of disease progression. The survival curves were built according to the Kaplan-Meier method, and the resulting curves were compared using the log-rank test. The joint effect of covariables was examined using the Cox proportional hazard regression model. For other analyses, the data are expressed as the mean ± standard deviation. Differences between groups were assessed using an unpaired, two-tailed Student’s t test; P < 0.05 was considered significant.

## Results

### Expression of miR-302b in ESCC and its significance

**Figure 1:**
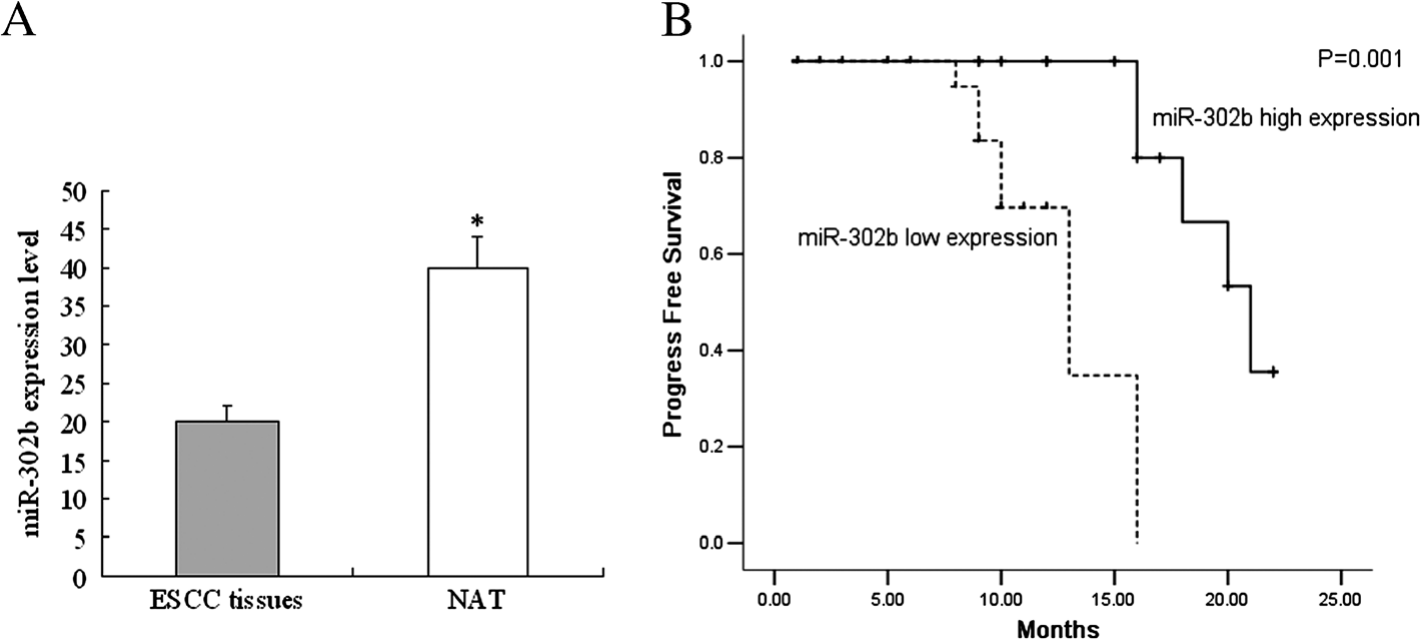
**Expression of ErbB4 in esophageal squamous cell carcinoma. A)** Relative expression of miR-302b expression levels in 50 surgical specimens of ESCC tissues and matched normal adjacent tissues (NAT) are shown. The data are presented as 2^-ΔCT^ values (*P < 0.05). **(B)** Patients with high miR-302b expression had a longer progression-free survival compared to patients with low miR-302b expression.

We examined the expression of miR-302b in a set of 50 paired samples using qRT-PCR. The results showed that miR-302b was significantly down-regulated in ESCC tissues when compared to the NAT (20 ± 3.42 *vs* 40 ± 5.24, P < 0.05, Figure 1A). Next, the correlation of miR-302b with the clinicopathological factors was examined. There was a correlation between the miR-302b expression status and the presence of lymph node metastases (Table 2). A low level of miR-302b expression and lymph nodes metastases correlated with a decreased progression-free survival (PFS) according to the Kaplan-Meier survival curve analysis with a log rank comparison; the other parameters were not significant (Table 3, Figure 1B). Decreased expression of miR-302b was an independent prognostic factor for PFS (Table 4).

**Table 2:** Clinicopathologic variables and the expression status of miR-302b

**Variables**	**N**	**miR-302b**		***P***
		**Low**	**High**	
Age				0.168
<65	34	21	13	
≥65	16	13	3	
Gender				0.863
Male	29	20	9	
Female	21	14	7	
Smoking				0.301
Yes	37	27	11	
No	13	7	6	
Drink				0.137
Yes	30	18	12	
No	20	16	4	
Differentiation				0.010
Well + Moderate	39	23	16	
Poor	11	11	0	
TNM stage				0.230
I–II	19	11	8	
III–IV	31	23	8	
Lymph node status				0.001
Metastasis	30	26	4	
No metastasis	20	8	12	

**Table 3:** Univariate analysis for progression free survival

**Variables**	**N**	**Progression free survival (months)**		***P***
		**Median ± SE**	**95% CI**	
miR-302b				0.001
Low	34	12.92 ± 1.03	10.91-14.93	
High	16	19.82 ± 0.77	18.32-21.33	
Age				0.676
<65	34	17.29 ± 1.23	15.28-19.31	
≥65	16	17.20 ± 2.63	12.05-22.35	
Gender				0.586
Male	29	17.26 ± 1.08	15.12-19.36	
Female	21	18.63 ± 1.45	15.78-21.47	
Smoking				0.173
Yes	37	16.37 ± 0.95	14.50-18.24	
No	13	18.94 ± 1.72	15.56-22.31	
Drinking				0.365
Yes	30	16.89 ± 1.15	14.63-19.15	
No	20	18.09 ± 1.17	15.80-20.39	
Differentiation				0.108
Well + Moderate	39	17.87 ± 1.00	15.91-19.83	
Poor	11	14.00 ± 2.54	9.20-18.80	
TNM stage				0.716
I–II	19	18.04 ± 1.22	15.65-20.43	
III–IV	31	16.79 ± 1.39	14.07-19.51	
Lymph node				0.005
Metastasis	30	14.67 ± 1.35	12.03-17.31	
No metastasis	20	20.2 ± 0.84	18.56-21.85	

**Table 4:** Multivariate Cox proportional hazards analysis for progression free survival

**Variables**	**Progression free survival**		***P***
	**HR**	**95% CI**	
miR-302b			
Low vs high	5.86	1.73-19.84	0.005
Lymph node			
Metastasis vs no metastasis	1.82	0.67-4.87	0.238
TNM stage			
III–IV vs I–II	1.25	0.57-2.72	0.583
Differentiation			
Well + moderate vs poor	0.89	0.31-2.54	0.826

### ErbB4 is a target of miR-302b

**Figure 2:**
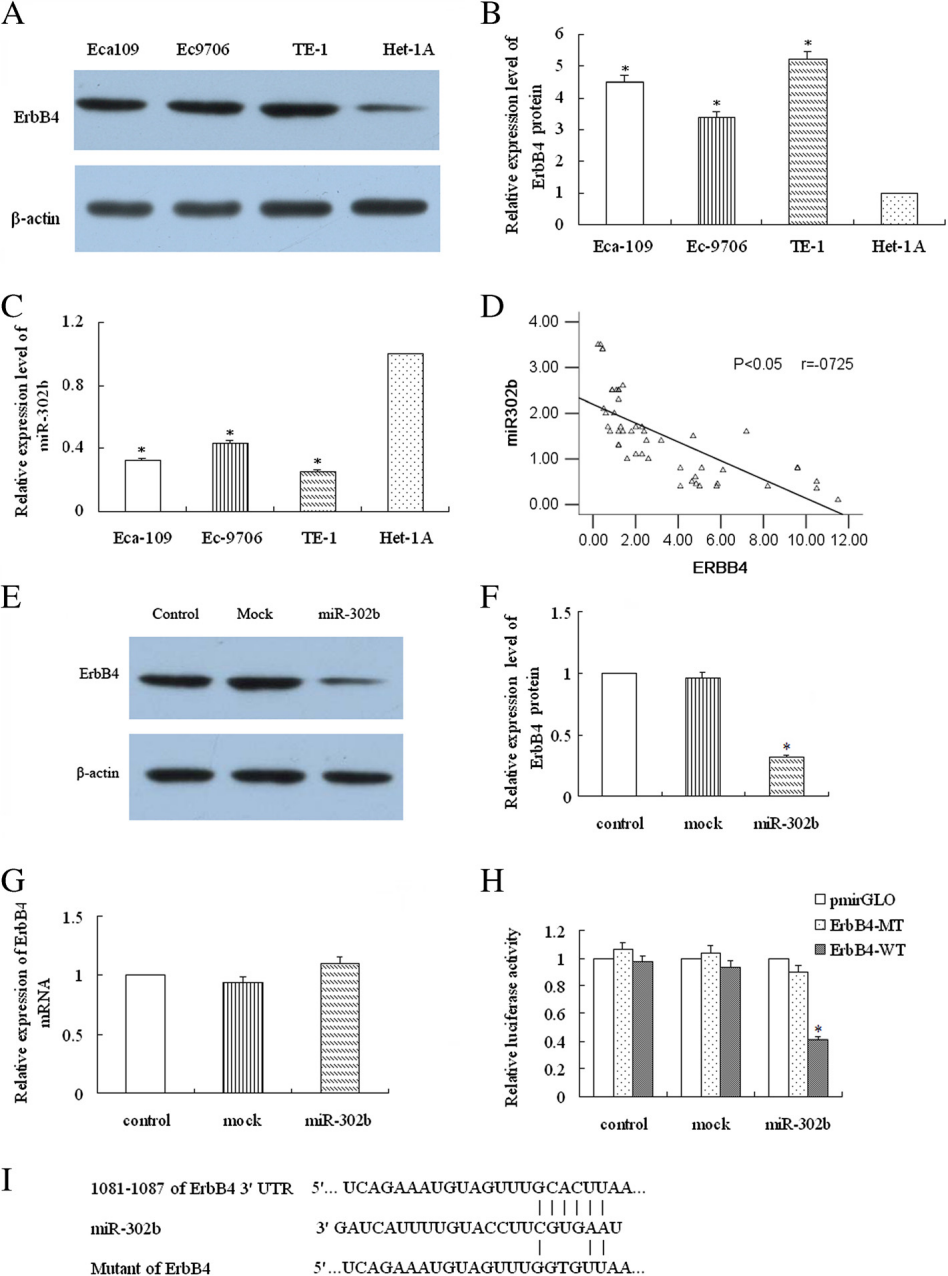
**miR-302b post-transcriptionally regulates ErbB4 expression. (A-B)** The expression of ErbB4 protein in ESCC cell lines (Eca109, Ec9706, and TE-1) and esaphagel normal cell line (Het-1A) were analyzed using immunoblot analysis. **(C)** The expression of miR-302b in three esophageal cancer cell lines and Het-1A were analyzed using RT-PCR. **(D)** The relationship between the miR-302b expression level and ErbB4 protein expression level in the specimens of the patients were analyzed. **(E-F)** The effect of miR-302b on ErbB4 protein expression was detected using immunoblot analysis in TE-1 cells. **(G)** The effect of miR-302b on the mRNA expression of ErbB4 was detected using qRT-PCR in TE-1 cells. **(H)** Luciferase reporter assay in TE-1 cells. **(I)** Diagram of the ErbB4 3*'*-UTR containing reporter constructs. “miR-302b” represents cells transfected with pcDNA™6.2-GW/EmGFP-miR-302b; “control” represents normal ESCC cells; “mock” represents cells transfected with pcDNA™6.2-GW/EmGFP-miR; “ErbB4-MT” and “ErbB4-WT” represent the mutated and wild type luciferase vectors, respectively. *P < 0.05 compared to control or mock respectively.

We first determined the expression levels of ErbB4 protein and miR-302b in three different esophageal cancer cell lines (Eca109, Ec9706, and TE-1) and one esaphagel normal cell line (Het-1A). We found that each cell line expressed higher level of ErbB4 protein and lower level of miR-302b than that in Het-1A (P < 0.05, Figure 2A, B, and C). Further, TE-1 had the highest expression levels of ErbB4 protein but lowest expression levels of miR-302b, while Ec9706 had the highest expression levels of ErbB4 protein but lowest expression levels of miR-302b. Therefore, we analyzed the relationship between the miR-302b expression level and ErbB4 protein expression level in the specimens of the patients. The result demostrated that miR-302b negatively correlated with the ErbB4 protein expression (Figure 2D, P < 0.05, r = −0.725). Then, TE-1 and Ec9706 were chosen for following experiments. After confirming that anti-miR-302b or miR-302b could significantly change the expression level of miR-302b using qRT-PCR, we then tested the effect of miR-302b on the expression of ErbB4 mRNA and protein. The results showed that miR-302b significantly decreased the expression of ErbB4 protein (P < 0.05, Figure 2E and F), but had no effect on mRNA expression (P > 0.05, Figure 2G). We next investigated whether the 3′-UTR of ErbB4 was a functional target of miR-302b in TE-1 cells. After co-transfection of miR-302b with either the ErbB4-wild type or mutated 3′-UTR luciferase reporter vector into TE-1 cells, we found that miR-302b reduced the activity of the luciferase reporter fused to the wild-type ErbB4 3′-UTR by 60%. However, mutation of the 3-nt sequence in the ErbB4 3′-UTR complementary to the miR-302b seed sequence restored the luciferase activity of the miR-302b transfected cells from 60% to 90%, showing that the action of miR-302b on ErbB4 depended on the presence of a single miR-302b cognate binding site within the 3′-UTR (Figure 2H and I).

### miR-302b represses cell proliferation by inducing apoptosis

**Figure 3:**
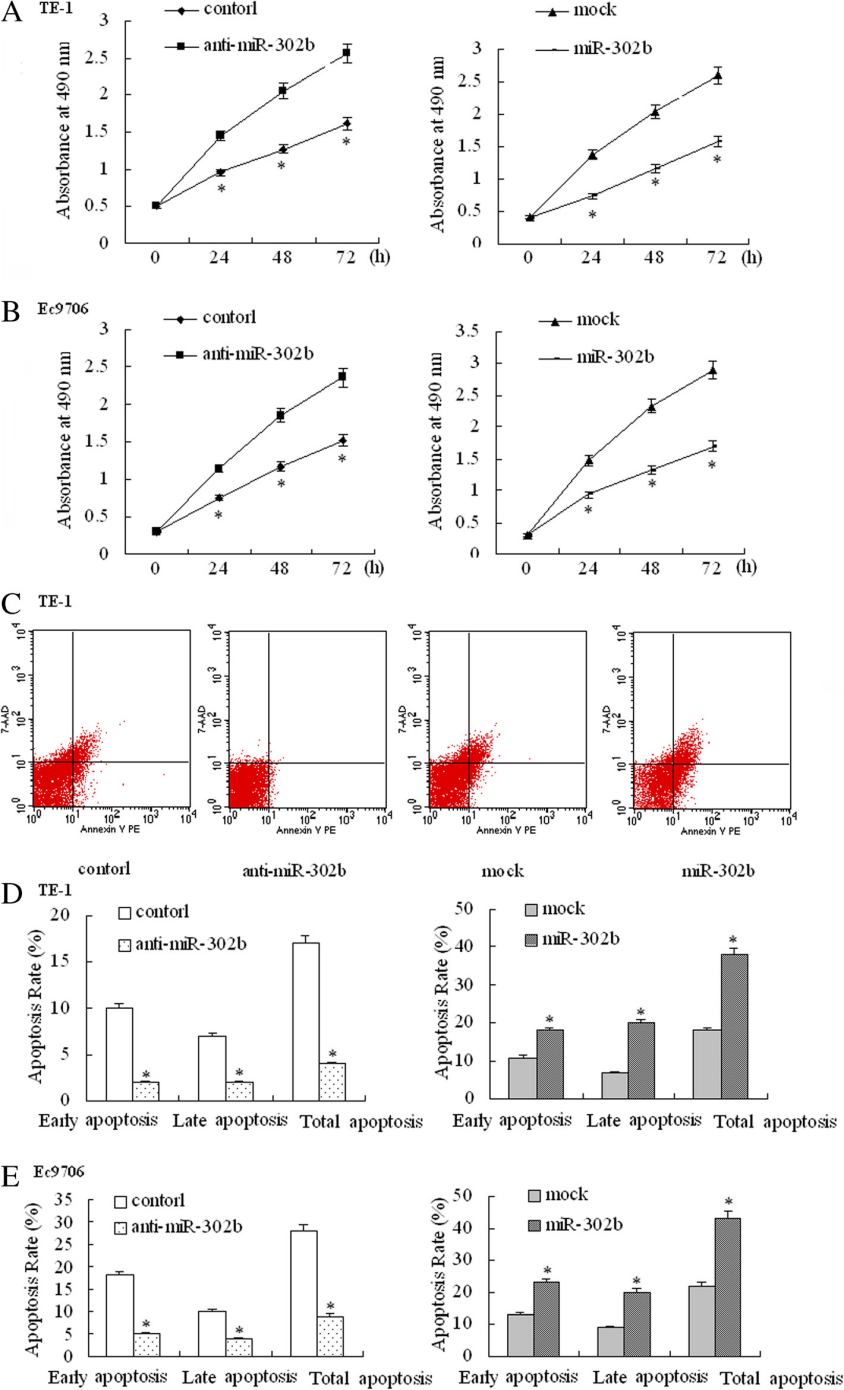
**Effect of miR-302b on cell proliferation and apoptosis. (A-B)** After pcDNA™6.2-GW/EmGFP-miR-302b (miR-302b) or Anti-miR-302b inhibitor (anti-miR-302b) transduction, the growth of TE-1 cells **(A)** and Ec9706 cells **(B)** was analyzed at different time points and compared to anti-miR-Inhibitors-Negative Control (control)/pcDNA™6.2-GW/EmGFP-miR (mock) cells using the MTT assay. **(C)** Flow cytometric analysis of the effect of miR-302b on apoptosis of TE-1 cells. **(D-E)** Flow cytometric analysis of the effect of miR-302b on the apoptosis of TE-1 cells **(D)** and Ec9706 cells **(E)**. *P < 0.05 compared with the respective control.

To investigate whether miR-302b modulates cell proliferation in esophageal cancer cells, we assayed its effect on cell proliferation activity. The proliferation activity of TE-1 and Ec9706 cells transfected with anti-miR-302b or miR-302b was determined using an MTT assay. As shown in Figure 3A and B, cells treated with anti-miR-302b had a significant increase in cell viability when compared with the anti-miR-NC transfected cells (P < 0.05). In contrast, overexpression of miR-302b resulted in a decrease in absorbance (P < 0.05). Further experiments demonstrated that this cell proliferation inhibition effect was partly due to the induction of apoptosis (Figure 3C,D and E). These results indicated that ESCC cell growth can be modulated through miR-302b-mediated ErbB4 repression.

### miR-302b regulates cell invasion *in vitro*

**Figure 4:**
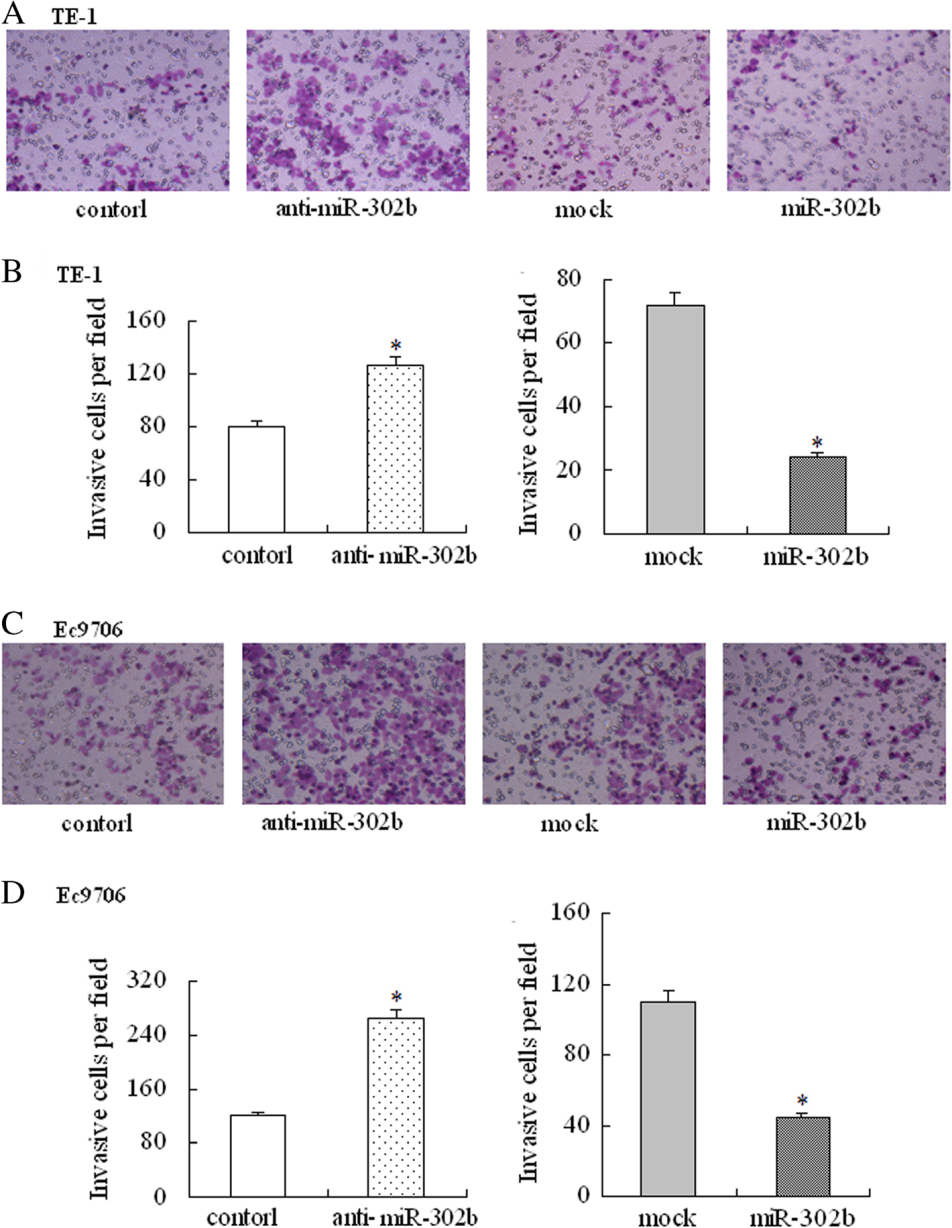
**Effect of miR-302b on cell invasion *in vitro. *(A-B)** Cells transfected with the anti-miR-302b inhibitor (anti-miR-302b), anti-miR-Inhibitors-Negative Control (control), pcDNA™6.2-GW/EmGFP-miR-302b (miR-302b), or pcDNA™6.2-GW/EmGFP-miR (mock) were subjected to transwell invasion assays. **(C-D)** The invasive cell numbers are the average count of five random microscopic fields detected using the transwell invasion assay. **A** and **C**: TE-1 cells; **B** and **D**: Ec9706 cells. Each bar represents the mean ± SD of the counts. *P < 0.05 compared with the respective control.

Because there was a correlation between miR-302b and lymph node metastasis, a transwell assay was performed to investigate the role of miR-302b on the invasion of ESCC cells. Overexpression of miR-302b repressed the cell invasion ability of TE-1 cells, while down-regulation of miR-302b expression had contrary results (P < 0.05, Figure 4A and B). The same result was also confirmed in Ec9706 cells. These findings suggest that miR-302b regulates cell invasion of the ESCC cell lines *in vitro*.

## Discussion

ErbB4 expression has been noted in various tumors, such as esophagus, colon, prostate, ovary, lung, breast, and thyroid [12-15,25-27]. Moreover, recent findings about somatic mutations that activate ErbB4 in metastatic melanoma have started to support a casual role of ErbB4 in carcinogenesis and to support the development of tools [28], such as ErbB4 antibodies, to target ErbB4 in cancer [29]. However, reports about the role of ErbB4 in ESCC are limited.

Previous studies have reported that miRNAs play important roles in gene expression regulation. However, the expression and the regulatory mechanisms of the ErbB4 gene in ESCC have not been reported. Using bioinformatics, ErbB4 was found to be a predicted target of miR-302b. miR-302b is a member of the miR-302 cluster, which is specifically expressed in pluripotent human embryonic stem cells but not in differentiated embryoid bodies or adult tissues [30]. This miR-302 family is also able to reprogram human skin cancer cells into a pluripotent ES cell-like state [22]. It was found that overexpression of miR-302b induced caspase-3-mediated apoptosis in the human neuroblastoma SH-SY5Y cell line [23]. But, a recent report found that miR-302b is overexpressed in primary human tumors specimens, and the down-regulation of miR-302b effectively decreased tumor cell growth in human head and neck squamous cell carcinoma patients [31].

Our results showed that miR-302b is down-regulated in tumor tissues compared to paired normal adjacent tissues. There were significant correlations between the expression of miR-302b and lymph node metastasis and differentiation. Furthermore, a low expression level of miR-302b was an independent factor that indicated poor prognosis in ESCC patients. This evidence suggests that down-regulation of miR-302b in tumor cells may play roles in the development of ESCC and may have prognostic value.

We then investigated whether ErbB4 could be regulated by miR-302b and the effect that miR-302b had on ESCC cell behaviors. Our study documented that ErbB4 protein expression was negatively regulated by miR-302b both in cell and tissue analysis. The overexpression of miR-302b significantly decreased the ErbB4 protein level but not mRNA level in ESCC cells, indicating the post-transcriptional down-regulation of ErbB4 by miR-302b. Moreover, the overexpression of miR-302b significantly decreased the luciferase activity of pmirGLO that contained the ErbB4 3′-UTR sequence, while it did not decrease the activity of pmirGLO that contained the ErbB4 3′-UTR mutant sequence, indicating that the target site was specific. Furthermore, to reveal the exact role of miR-302b in ESCC, we tested the effect of miR-302b on proliferation, apoptosis, and invasion by up- and down-regulating the expression level of miR-302b. The results suggested that miR-302b acted as a tumor suppressor gene in ESCC by inhibiting proliferation, inducing apoptosis, and repressing invasion. Contrary to our observations, Murray et al. showed that miR-302b was overexpressed in malignant germ cell tumors compared to normal gonad and benign germ cell tumors [32]. But miR-302b function as a tumor suppressor gene both in gastric cancer by targeting EGFR [33]. These results indicate that onco-miRNAs and suppressor-miRNAs can regulate two different roles of the same gene, behaving as oncogenes or tumor suppressors, depending on the tissue type and specific targets [34]. We will carry out further *in vivo* experiments to confirm the role of miR-302b and its target genes in ESCC.

## Conclusions

This was the first study to evaluate the relationship between ErbB4 and miR-302b in ESCC. Our findings demonstrated that ErbB4 was up-regulated in ESCC, and expression of ErbB4 was correlated with tumor differentiation and lymph node metastasis. We found that miR-302b post-transcriptionally down-regulated ErbB4 expression *in vitro*. We also concluded that miR-302b inhibited proliferation by inducing apoptosis and repressed the invasive ability of ESCC cells, and an ErbB4-mediated pathway may be involved in this function.

## Competing interests

The authors declare that they have no competing interests.

## Authors’ contributions

MZ, LZ and QY constructed the manuscript. MZ, LZ and QY were responsible for clinical data and evaluated clinical data; formed analysis of relation between clinical data and survival data. QY, SZ and WY carried out intro experiments. ZL, CH, QW, and JW reviewed the manuscript. All authors read and approval the final manuscript.
